# Impact of Oral Care and Antisepsis on the Prevalence of Ventilator-Associated Pneumonia

**DOI:** 10.3290/j.ohpd.a44443

**Published:** 2020-07-04

**Authors:** Luciana F Galhardo, Gilson F Ruivo, Fernanda O Santos, Tamires T Ferreira, Juliana Santos, Mariella VP Leão, Debora Pallos

**Affiliations:** a Researcher, Department of Dentistry, University of Taubaté, Taubaté, São Paulo, Brazil. Performed the experiments.; b Assistant Professor, Department of Medicine, University of Taubaté, Taubaté, São Paulo, Brazil. Study idea, hypothesis; experimental design; proofread the manuscript.; c Researcher, University of Taubaté, Taubaté, São Paulo, Brazil. Performed the experiments.; d Researcher, University of Taubaté, Taubaté, São Paulo, Brazil. Performed the experiments.; e Researcher, University of Taubaté, Taubaté, São Paulo, Brazil. Performed a certain test.; f Assistant Professor, Basic Bioscience Institute, University of Taubaté, Taubaté, São Paulo, Brazil. Study idea, hypothesis, experimental design, wrote the manuscript, proofread the manuscript.; g Assistant Professor, University of Santo Amaro – UNISA, Santo Amaro, São Paulo, Brazil. Study idea, hypothesis, experimental design, wrote the manuscript, proofread the manuscript.

**Keywords:** chlorhexidine, mechanical ventilation, oral care, pneumonia

## Abstract

**Purpose:**

This study aimed to evaluate the impact of oral care and use of chlorhexidine gluconate on the prevention of ventilator-associated pneumonia (VAP) in patients admitted to an intensive care unit (ICU).

**Materials and Methods:**

An evaluation was performed on 229 patients admitted to ICU in 2012 (before implementation of oral care protocol) and 329 in 2013 (after the protocol). Oral care was based on the removal of secretions from the oral cavity with 0.12% chlorhexidine solution for brushing and sterile gauze for cleaning before a new aspiration. The cases of VAP were evaluated by observing respiratory signs, radiological changes, and culture and laboratory results. The following data were also analysed: gender, length hospital of stay, mechanical ventilation, use of antibiotics and aetiological agent of infection.

**Results:**

There was a tendency towards lower risk of development of VAP after application of oral care protocol (odds ratio = 0.64–95% CI: 0.39–1.04). There was also a reduction in the incidence of early pneumonia (up to 72 h of hospitalisation). With regard to the aetiological agent of infections, although Gram-negative bacteria predominated in the two periods studied, there was a decrease in the cases of *Staphylococcus aureus* infection.

**Conclusion:**

Oral care protocol has statistically significantly reduced the risk of developing early VAP in ICU patients, thus demonstrating the importance of multidisciplinary teamwork for hospitalised patients.

Mechanical ventilation (MV) aims to reverse or prevent respiratory muscle fatigue, as well as to reduce its workload, to decrease oxygen consumption and to maintain gas exchanges. Thus, it also reduces respiratory discomfort and allows application of specific therapies.^3^

The use of MV in the intensive care unit (ICU) is frequent, but exposes patients to the risk of acquiring pneumonia. The ventilator-associated pneumonia (VAP) is defined as a pneumonia occurring 48–72 h or after endotracheal intubation, being characterised by the presence of new or progressive infiltrate, signs of systemic infection (ie, fever, altered white blood cell count), changes in sputum characteristics and detection of a causative agent. In the ICU, the incidence of VAP occurs in 9–27% of the orotracheal intubations.^1,2^

A multidisciplinary strategy for prevention of VAP is recommended.^11^ Most of the studies aimed to achieve a zero rate of VAP, excluding high-risk subjects such as those who were immune compromised.^19^

Pena-López et al^19^ implemented a care bundle to prevent ventilator-associated infections (VAIs) in children, reporting reduction in the VAP rate and delay in the onset of ventilator-associated tracheobronchitis (VAT). Although tracheostomised children were at increased risk of VAI, preventive measures had a greater impact on them.

Poor oral care has been linked to higher incidence of VAP.^15^ Marino et al^13^ observed that patients admitted to adult intensive care had poor oral health, which improved after oral care with toothbrush or foam swab. Both interventions were equally effective in removing plaque and reducing gingival inflammation.

Toothbrushing twice a day with distilled water reduced the incidence of VAP in patients admitted to the ICU. Therefore, it has been recommended that nurses caring for ventilator-dependent patients brush their teeth with distilled water as part of a routine oral care.^16^

The aim of this study is to verify the impact of oral hygiene and use of chlorhexidine gluconate on the prevention of VAP in patients admitted to an ICU. The null hypothesis is that the use of chlorhexidine gluconate does not influence oral hygiene and the prevention of VAP in patients admitted to an ICU.

## Materials and Methods

### Study Population

A descriptive, retrospective study was carried out on patients admitted to the ICU of the Regional Hospital of Vale do Paraíba, Taubaté, São Paulo, Brazil, which is a reference hospital for severe diseases. A total of 229 patients were evaluated in 2012 (before implementation of an oral care protocol with chlorhexidine) and 329 patients in 2013 (after protocol).

The present study was performed according to the Declaration of Helsinki and approved by the UNITAU Research Ethics Committee (process number 23516813.4.0000. 5501) – PH/CEP.

### Oral Care Protocol

In 2013, all ICU patients began receiving oral care by means of a protocol implemented in the Regional Hospital of Vale do Paraíba as follows.

Initially, the oral cavity was aspirated with a sterile aspiration probe before being brushed with chlorhexidine gluconate (0.12%) and disposable toothbrushes. Next, a sterile gauze soaked in saline solution was used to clean the oral mucosa. Finally, the remaining salivary content of the oral cavity was aspirated again. These procedures were performed by the same team of nursing assistants under supervision and training of the same dental practitioner.

### Recorded Parameters

Data from each patient were collected from either electronic records or databases of the clinical analysis laboratories.

The following data were recorded: gender, reason and length of hospitalisation, length of MV, need of tracheostomy, presence of VAP, time of VAP occurrence, VAP aetiology, use of antibiotics (ie, type, length of use, changes after development of pneumonia) and occurrence of death.

The patients were diagnosed as having VAP according to the following criteria: respiratory signs (presence of pulmonary secretion and level of respiratory discomfort); radiological alteration (new pulmonary infiltrate on chest radiograph or worsening of pre-existing lung injury); cultures (positive blood culture and/or positive culture of pulmonary secretion, and if possible, isolation of the aetiological agent); laboratory data – alterations on leukogram, CRP values and/or arterial blood gas analysis (ie, calculation of the relationship between arterial oxygen pressure and fraction of oxygen used by the patient to determine whether the patient is suffering severe hypoxia or not).

VAP is an important cause of infection in ICU patients who need invasive MV with endotracheal tube or tracheostomy and can be classified into two groups according to the length of MV: early onset (within the first 96 h) or late onset (after 96 h).^10^ Tracheostomy was performed in patients undergoing prolonged MV according to the institutional protocol. Non-invasive sampling using endotracheal or tracheostomy aspiration with semiquantitative culture was the laboratory method to diagnose VAP.^23^ VAP aetiology was obtained from databases of the clinical analysis laboratories and the methodology of microbial identification was in agreement with their protocols.

### Statistical Analysis

Categorical variables were described as frequencies (%) and the normally distributed variables were described as mean value ± standard deviation. For comparison of the continuous variables between the two groups, Student’s test was used for normally distributed data and Mann–Whitney U test for non-normal data. Comparative analysis of categorical variables was performed by using one-way analysis of variance (ANOVA) and/or Chi-squared tests. Data obtained in the periods before and after implementation of the oral care protocol were compared by using odds ratio test and statistical analysis. The statistical significance level adopted in all analyses was 5% (p ≤ 0.05) and the statistical software GraphPad Prism 6.0 was used.

## Results

By comparing the prevalence of VAP in 2012 and 2013, a reduction was observed in the percentage of VAP development after the implementation of oral care protocol with chlorhexidine. Of the 229 patients admitted to the ICU in 2012, 38 (16.59%) developed VAP. In 2013, of the 329 patients, 37 (11.25%) developed VAP. By applying the odds ratio test = 0.64 (95% CI: 0.39–1.04), it was observed that there was a tendency towards lower risk of development of VAP after application of oral care protocol.

Those patients who developed VAP in 2012 and 2013 were mostly male, that is, 25 (65.79%) male and 24 (64.86%) females, respectively.

With regard to mean age of the patients, length of hospital stays and use of antibiotics, it was observed that there were no statistically significant differences before and after implementation of the oral care protocol (Table 1).

**Table 1:** Comparison of patients hospitalised in ICUs in the years of 2012 and 2013 diagnosed with ventilator-associated pneumonia (VAP), showing mean age, length of hospitalisation, amounts and length of antibiotic use

	2012	2013	p
	n = 38	n = 37	
Age	48.89 ± 18.65	49.51 ± 21.03	0.9010
Length of hospitalisation	26.74 ± 18.65	31.77 ± 24.30	0.2574
Antibiotic amounts	3.42 ± 1.20	3.89 ± 1.05	0.1119
Length of antibiotic use	17.53 ± 10.13	17.97 ± 6.10	0.3057

In 2012, of the 38 patients with VAP, 27 (71.05%) were discharged from the ICU and 11 (28.95%) died. In 2013, of the 37 who had VAP, 23 (62.16%) were discharged and 14 (37.84%) died. There was no statistically significant difference between the cases of deaths (X2 p = 0.4142).

By comparing the data of patients who were discharged to those of patients who died, no statistically significant differences were found between 2012 (age: p = 0.0956, length of hospital stay: p = 0.9917, number of antibiotics: p = 0.0814; length of use: p = 0.2267) and 2013 (age: p = 0.1495, length of hospital stay: p = 0.1619, number of antibiotics: p = 0.3164; length of use: p = 0.1840). Likewise, no statistically significant differences were observed when one compared data of patients who were discharged from hospital in 2012 and 2013 (age: p = 0.8817, length of hospital stay: p = 0.9917, number of antibiotics: p = 0.1014, time of use: p = 0.9197) and data of patients who died in 2012 and 2013 (age: p = 0.8835, length of hospital stay: p = 0.1978, number of antibiotics: p = 0.7723, length of use: p = 0.1637).

By analysing the period of development of VAP, it was observed that there was a reduction in the number of early onset pneumonia in 2013. In 2012, of the 38 patients, six (15.79%) occurred early, whereas in 2013, of the 37 patients, only three (8.11%) occurred prematurely (p = 0.3258)

No patient admitted to the ICU in 2012 required a tracheostomy, whereas nine (24.32%) patients received this procedure in 2013.

With regard to the microorganisms present in the biological samples of the patients, it was possible to identify the aetiological agent in 29 samples in 2012 and in 36 samples in 2013. In the early period of the implementation of oral care, the number of individuals with polymicrobial infection (caused by more than one microorganism) was one, whereas in the later period 10 individuals presented polymicrobial infection (data not shown). There was also a change in the aetiological profile of the infections (Table 2). Although *Acinetobacter* remained the most isolated aetiological agent in the two periods of study, there was a decrease in the cases of *Staphylococcus aureus* infection and an increase in infections caused by *Klebsiella pneumonia*, *Pseudomonas* and coagulase-negative *Staphylococcus*.

**Table 2:** Change in the aetiological profile of infections (c2 test)

Aetiological agent	2012	2013	p
	Identified = 30	Identified = 46	
*Acinetobacter*	9 (30.0%)	16 (34.78%)	0.7570
*Klebsiella pnemoniae*	5 (16.67%)	13 (28.26%)	0.3560
*Pseudomonas*	1 (3.33%)	6 (13.04%)	0.1874
*Escherichia coli*	1 (3.33%)	0 (0%)	–
Gram-negative	2 (6.67%)	1 (2.17%)	0.3467
TOTAL GRAM-NEGATIVE	18 (60%)	36 (78.26%)	0.3134
			
*Staphylococcus aureus*	6 (20%)	3 (6.52%)	0.1185
Coagulase-negative* Staphylococcus*	4 (13.33%)	6 (13.04%)	0.9745
Gram-positive	1 (3.33%)	0 (0%)	–
TOTAL GRAM-POSITIVE	11 (36.67%)	9 (19.57%)	0.7987
			
*Candida*	1 (3.33%)	1 (2.17%)	0.7640

## Discussion

The insertion of dentistry in the hospital setting is already a reality. Colonisation of the oral microenvironment by pathogenic microorganisms is extremely relevant in the pathophysiology of pneumonia, especially in patients with low consciousness. In up to 70% of the cases, this condition favours the microaspiration of oropharyngeal secretions.^3^ The incidence of pulmonary infections in intubated patients undergoing MV ranges between 9% and 27%.^2^ In the present study, the incidence of VAP ranged from 11.25% to 16.59%, thus corroborating the literature.

Several studies have already evaluated the impact of professional dental care on the clinical conditions of hospitalised patients. It has been observed that oral decontamination is effective in preventing VAP and reducing the incidence of oral microorganisms such as respiratory pathogens^4,12,20,22^ independently of the methodology of the study.

The present study has confirmed these findings as there was a reduction in the prevalence of VAP after implementation of the oral hygiene protocol with 0.12% chlorhexidine in patients admitted to the ICU. It is important to note that the population investigated before and after the implementation of this protocol was similar in terms of age, length of stay and antibiotic therapy.

The results have also shown that in 2013 the number of deaths was higher despite the lower prevalence of VAP, suggesting a greater severity of this pathology or a worsened condition of the patients in this period. In fact, in 2013 there were more cases of late VAP, generally related to prolonged periods of intubation and consequent need of tracheostomy. Early onset VAP is usually less severe, with a better prognosis and more likely to be caused by antibiotic-sensitive bacteria. On the other hand, late onset VAP is usually caused by multidrug resistant pathogens and is associated with increased morbidity and mortality.^6,8^ A greater number of polymicrobial infections were also observed in 2013, which is compatible with a worse prognosis, although most of the cases of VAP have been recognised as being of polymicrobial nature.^21^

With regard to the aetiology, *Acinetobacter* and Gram-negative bacteria have remained more frequent despite the considerable reduction in the cases of *S. aureus* VAP. *Acinetobacter* spp., *Pseudomonas* spp., *Klebsiella pneumonia* and *Staphylococcus aureus* are common VAP pathogens, all identified with varying prevalence. As mentioned before, up to 40% of these infections can be polymicrobial.^7,8,18^ The reduction of *S. aureus* infections coincides with the decrease in the development of early pneumonia in 2013. In the same way, Joseph et al^9^ found that *S. aureus* was more common in early onset VAP and that *Pseudomonas* spp. and *Acinetobacter* spp. were significantly associated with late onset VAP, with many of them being multidrug resistant pathogens.^5,9^

Studies show that after 48 h of ICU admission, all patients present formation of oropharyngeal biofilm (especially by Gram-negative bacteria), which is considered a reservoir of respiratory pathogens.^4^ For this reason, our oral care protocol aimed at using not only chlorhexidine as an antimicrobial agent, but also a toothbrush for the mechanical removal of any biofilm.

## Conclusion

The results of the present study have confirmed the great importance of a dental professional together with the multidisciplinary team in order to perform preventive and curative activities for promotion of oral health, thus improving the general clinical picture of the hospitalised patient. These results were also verified by Özçaka,^17^ who found a reduced incidence rate of VAP with the use of chlorhexidine gluconate in ICU patients.

The prevention of VAP should follow a multidirectional approach by means of protocol interventions developed to reduce the colonisation of the bacteria in the oral cavity and nasopharynx. Therefore, a systematic implementation of a multidisciplinary team approach can reduce the incidence of VAP. A further sustained improvement requires persistent vigilant inspections.^14^ With the expansion of the dentist’s role in the prevention of VAP, a new horizon opens for the development of new activities in a highly complex and unexplored field for the dental professional.
